# Acute and Chronic Plasma Metabolomic and Liver Transcriptomic Stress Effects in a Mouse Model with Features of Post-Traumatic Stress Disorder

**DOI:** 10.1371/journal.pone.0117092

**Published:** 2015-01-28

**Authors:** Aarti Gautam, Peter D’Arpa, Duncan E. Donohue, Seid Muhie, Nabarun Chakraborty, Brian T. Luke, Dmitry Grapov, Erica E. Carroll, James L. Meyerhoff, Rasha Hammamieh, Marti Jett

**Affiliations:** 1 US Army Center for Environmental Health Research, Fort Detrick, MD, United States of America; 2 The Geneva Foundation, Tacoma, WA 98402, United States of America; 3 Advanced Biomedical Computing Center, Frederick National Laboratory for Cancer Research, Frederick, MD, United States of America; 4 NIH West Coast Metabolomics Center, University of California Davis, Davis, CA, United States of America; 5 Army Institute for Public Health, Aberdeen Proving Ground, Aberdeen, MD 21010–5403, United States of America; Technion—Israel Institute of Technology, ISRAEL

## Abstract

Acute responses to intense stressors can give rise to post-traumatic stress disorder (PTSD). PTSD diagnostic criteria include trauma exposure history and self-reported symptoms. Individuals who meet PTSD diagnostic criteria often meet criteria for additional psychiatric diagnoses. Biomarkers promise to contribute to reliable phenotypes of PTSD and comorbidities by linking biological system alterations to behavioral symptoms. Here we have analyzed unbiased plasma metabolomics and other stress effects in a mouse model with behavioral features of PTSD. In this model, C57BL/6 mice are repeatedly exposed to a trained aggressor mouse (albino SJL) using a modified, resident-intruder, social defeat paradigm. Our recent studies using this model found that aggressor-exposed mice exhibited acute stress effects including changed behaviors, body weight gain, increased body temperature, as well as inflammatory and fibrotic histopathologies and transcriptomic changes of heart tissue. Some of these acute stress effects persisted, reminiscent of PTSD. Here we report elevated proteins in plasma that function in inflammation and responses to oxidative stress and damaged tissue at 24 hrs post-stressor. Additionally at this acute time point, transcriptomic analysis indicated liver inflammation. The unbiased metabolomics analysis showed altered metabolites in plasma at 24 hrs that only partially normalized toward control levels after stress-withdrawal for 1.5 or 4 wks. In particular, gut-derived metabolites were altered at 24 hrs post-stressor and remained altered up to 4 wks after stress-withdrawal. Also at the 4 wk time point, hyperlipidemia and suppressed metabolites of amino acids and carbohydrates in plasma coincided with transcriptomic indicators of altered liver metabolism (activated xenobiotic and lipid metabolism). Collectively, these system-wide sequelae to repeated intense stress suggest that the simultaneous perturbed functioning of multiple organ systems (e.g., brain, heart, intestine and liver) can interact to produce injuries that lead to chronic metabolic changes and disorders that have been associated with PTSD.

## Introduction

Post-traumatic stress disorder (PTSD) is a commonly occurring, debilitating anxiety disorder that often persists for many years and is frequently associated with exposure to multiple traumas [1]. Significant controversy surrounds the diagnosis of PTSD [2] that is based on history of exposure to extreme traumatic event(s) and reported symptoms including persistent re-experiencing of the event(s), persistent avoidance and numbing to trauma-related stimuli, increased arousal, and clinically significant distress that impairs social, occupational, or other important areas of functioning for more than one month [3]. The PTSD lifetime prevalence rate from the National Comorbidity Survey was estimated at 7.8% among the general population of American men and women [4]. PTSD prevalence rates are higher in individuals exposed to severe traumas, and in post-conflict settings ranged from 16 to 37% [5,6] with 22% of 289,328 veterans of the Iraq and Afghanistan conflicts entering VA health care from 2002 to 2008 after having received the diagnosis of PTSD [7].

Neurological/physiological alterations associated with PTSD comprise both central and autonomic nervous systems, and include hyperarousal of the sympathetic nervous system with sensitized and augmented acoustic-startle eye blink reflex and sleep abnormalities, as well as changes in noradrenergic, hypothalamic-pituitary-adrenocortical, serotonergic, glutamatergic, thyroid, endogenous opioid, and other systems [6]. Brain imaging has revealed reduced volume of the hippocampus and anterior cingulate cortex, excessive amygdala activity, and reduced activation of the prefrontal cortex.

Psychometric and psychophysiologic techniques have been developed to assess PTSD [8]. Self-reporting comprises a large part of the diagnosis of PTSD, but it is prone to controversial diagnoses. Additional controversy in the diagnosis of PTSD relates to the high rate of comorbid conditions; i.e., individuals who meet the PTSD diagnostic criteria are likely to meet criteria for one or more additional diagnoses [6,9]. As such, PTSD may be categorized into multiple dimensions with traits such as cognition, mood, and social interactions existing on a continuum from normal to extreme [10]. Ongoing research aims to continue to link these behavioral dimensions with underlying molecular pathologies in order to construct valid, measurable traits for PTSD as well as to clarify the boundaries and overlap with other mental disorders, toward improving PTSD prevention, diagnosis and treatment [10].

Animal models of anxiety disorders vary widely. In the current study, we used a previously validated C57BL/6J mouse model with PTSD-like features [11]. Mice are stressed by exposures to trained aggressor mice for 5 or 10 6-hr sessions daily in a cage-in-cage environment wherein the subject mouse is removed from the smaller cage for up to three random times during each 6 hr session to be attacked by the aggressor for one minute or ten strikes, whichever comes first. In previous studies with this model, subject mice experienced acute stress effects, including body weight gain, increased body temperature, cardiac inflammation and transcriptomic changes, and behaviors indicative of fear and anxiety. Some of these acute stress effects persisted following 1.5 to 6 weeks of stress-withdrawal, reminiscent of PTSD. Here we have evaluated additional acute and chronic stress effects in this model, including changes in the transcriptome of liver and proteins and metabolites in plasma. In particular, transcriptomics indicated liver inflammation at 24 hrs post-stressor. Unbiased metabolomics identified altered metabolites derived from the gut microbiota as early as 24 hrs post-stressor that remained altered up to 4 wks. Also, at this chronic time point, aggressor-exposed mice displayed metabolomic markers of hyperlipidemia and transcriptomic indicators of altered xenobiotic and lipid metabolism in liver. These observations contribute to defining acute stress effects and their chronic sequelae following repeated exposures to intense stressors.

## Results and Discussion

### Study Design

**Figure 1:**
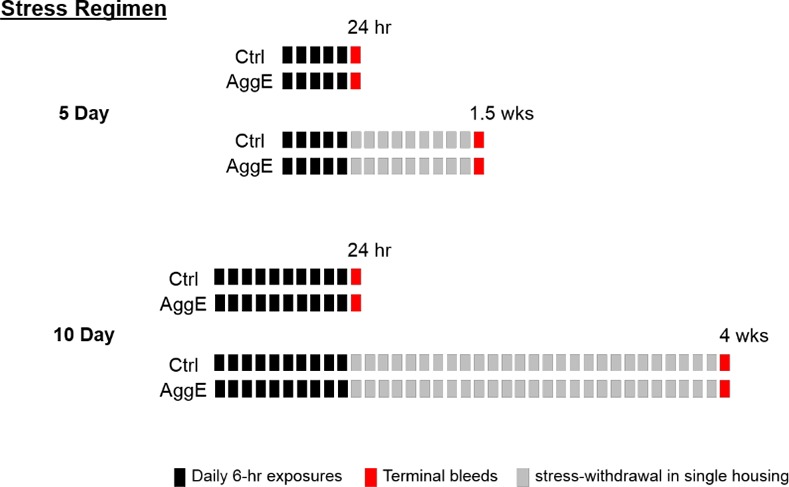
Study design. Mice were exposed to a trained aggressor mouse for 6 hrs daily for 5 or 10 days (black rectangles). Control mice were exposed on the same schedule and under the same conditions but with no aggressor mouse present. Terminal bleeds and tissue samples (red rectangles) were obtained 24 h after the 5- and 10-day regimens as well as 1.5 weeks after the 5-day and 4 weeks after the 10-day stressor regimens. Mice were singly housed throughout the experiment.

C57BL/6J mice were analyzed for unbiased plasma metabolomics, serum proteins, histopathology, and liver transcriptomics following control (Ctrl) or aggressor exposure (AggE) for 6 hrs daily for either 5 or 10 days. Twenty-four hours after these stressor regimens, as well as 1.5 weeks after the 5-day regimen, and 4 weeks after the 10-day regimen, histopathology was performed and terminal bleeds and tissues were obtained for analysis. One hour prior to sample collection, during a partition test [11], mice were exposed to the potent situational cue associated with the aggressor exposures. The experimental design is shown in Fig. 1.

### Plasma metabolite alterations of AggE mice at 24 hrs post-stressor partially normalized after stress-withdrawal for 1.5 or 4 wks

The unbiased plasma metabolomics detected 330 named metabolites and 166 unidentified compounds (S1 File). For the named metabolites, the pairwise comparisons of their levels between Ctrl and AggE were associated with high *q*-values, likely due to the small sample size of 5 or 6 mice per group and the known highly variable response of even inbred mice to stress. However, analysis of unadjusted p-values showed trends in the data. Clearer trends were identified by analyzing superpathways and subpathways of metabolites constructed based on KEGG, HMDB and the internal knowledge base of Metabolon, Inc. (Durham, NC). Within many of these pathways, numbers of elevated or suppressed metabolite levels in AggE vs. Ctrl differed from the null hypothesis of equal numbers (binomial test).

In pairwise analysis, AggE-altered metabolites were more numerous at 24 hrs after the last stress session as compared to after 1.5 or 4 wks of stress-withdrawal (Welch’s t-test and 2-way ANOVA): 40 and 14 metabolites were altered at 24 hrs and 1.5 wks, respectively (*p* ≤ 0.05), after the 5-day stress regimen; and, 37 and 20 metabolites were altered at 24 hrs and 4 wks, respectively (*p*≤ 0.05), after the 10-day stress regimen. This trend suggests that the acute metabolic perturbation of AggE mice partially normalized toward the Ctrl level after the mice had been withdrawn from the stressor for 1.5 and 4 wks.

### Evidence for habituation to the 10-day vs. the 5-day stressor regimen

Time-dependent changes in plasma metabolites were also evident in both Ctrl and AggE mice. In AggE mice, 103 (5-day regimen) and 30 (10-day regimen) metabolite levels were significantly different between the acute (24 hrs) and chronic time points (1.5 or 4 wks). Similarly, in control mice, 90 (5-day regimen) and 76 metabolites differed (10-day regimen) between the acute and chronic time points (S1 Table). Thus, greater numbers of metabolites were altered with time after the 5-day regimen as compared to the 10-day regimen, despite the 10-day-regimen mice having received five additional AggE sessions and having been withdrawn from the stress for 2.5 wks longer than the 5-day-regimen mice. This may indicate that the mice receiving the 10-day regimen habituated to the stressor between the 5^th^ and 10^th^ daily exposure (i.e., metabolites had already normalized 24 hrs after the 10^th^ stressor session). Consistent with this interpretation, the treatment effect (Ctrl vs. AggE, acute and chronic time points combined), the time effect (acute vs. chronic; Ctrl and AggE combined), and the treatment and time interaction (two-way ANOVA), were all greater after the 5-day compared to the 10-day stress regimen (S1 Table). Thus, there were fewer metabolic changes 24 hrs after the 10-day stress regimen as compared to the 5-day stress regimen, suggesting habituation to the metabolic effect of the stressor.

Consistent with habituation, the percent of mice that jumped in response to the aggressor or fought back against the aggressor dropped between the 4^th^ and 10^th^ days of the stress regimen (unpublished data). Additionally, in the heart, an order of magnitude fewer AggE-vs.-Ctrl transcripts were evident at 24 hrs after the 10-day than after the 5-day regimen, and inflammation transcripts peaked on the third day of the AggE regimen and then fell as tissue remodeling transcripts rose, suggesting healing despite the 6 hr daily AggE sessions on days 4 to 10 [12].

As a further indication of habituation, in the liver there were ~3-fold more AggE-vs.-Ctrl differentially expressed genes (DEGs, > 2-fold) at 24 hrs after the 5-day stressor than at 24 hrs after the 10-day stressor (2169 vs. 668 for 5- and 10-day mice, respectively; Table 1). Gene set enrichment analysis of these DEGs showed the downstream functions ‘biosynthesis of hydrogen peroxide’, ‘accumulation of leukocytes’, and ‘activation of phagocytes’ as the top three and only activated functions at 24 hrs post-5-day stressor (category ‘Physiological System Development and Function’, (S2 Table). Conversely, at 24 hrs after the 10-day stressor, the top activated function was ‘binding of fibroblast cell lines’, and the top two inhibited functions were ‘quantity of neutrophils’ and ‘quantity of phagocytes’. These results are consistent with those from heart tissue (below and [12]) and suggest that inflammation of livers of AggE mice at 24 hrs post-5-day stressor may have transitioned to fibroblast infiltration at 24 hrs post-10-day stressor.

**Table 1:** Summary of differentially expressed genes between livers of Control and AggE mice.

	5-Day Stress Regimen		10-Day Stress Regimen	
	24 hr	1.5 wk	24 hr	4 wk
Up-regulated genes^*^	842	530	383	193
Down-regulated genes^*^	1327	522	285	139
Totals	2169	1052	668	332

^*^ Based on ≥ 2 fold change of probe intensity with *p*-value ≤ 0.05.

### Heart histopathology was unique to AggE mice; lymphoplasmacytic infiltrates of other organs were similar in Ctrl and AggE mice but varied with stressor duration and post-stress time

Similar to the heart transcriptomics, histopathology of heart tissue of AggE mice at 24 hrs after the 5-day stress regimen appeared to be a higher grade than after the 10-day regimen [11]. Heart inflammatory histopathologies of the 5-day-AggE mice at 24 hrs had disappeared by 1.5 wks when fibrosis or fibroplasia and myocardial degeneration were present. In 10-day AggE mice at the 24 hr point, much more fibrosis, fibroplasia and degeneration were present compared to at 24 hrs after the 5-day AggE, indicating the injury had started to heal by the 11^th^ day after the start of the 10-day regimen. A larger percentage of 5-day AggE mice at 1.5 wks than the 10-day AggE mice at 4 wks showed fibrosis or fibroplasia and myocardial degeneration, which are likely remnants of the injury that persisted at 1.5 wks but was evidently healed by 4 wks (S1 Fig.).

Acute, intense psychological stressors such as inter-male aggressive encounters or catecholamine administration have been shown to induce organ tissue injury [13,14]. Upon fighting, Epidermal Growth Factor (EGF) released from submandibular salivary glands protected against heart injury, but not against liver injury [15]. However, twice daily adrenergic stimulation decreased the EGF content of submandibular glands by almost 90%, and further adrenergic stimulation did not increase plasma EGF levels [15]. This result suggests protective mechanisms against acute stress can become exhausted in response to chronic stress. In our model, the repeated, intense, 5- and 10-day AggE sessions likely exhausted mechanisms for protecting heart tissue from intense adrenergic stimulation.

In contrast to the cardiac lesions found only in AggE mice, lymphoplasmacytic infiltrates of liver, gallbladder, kidney, and stomach were of similar grades in Ctrl and AggE mice. This suggests the possibility that the Ctrl condition, which reproduced the AggE condition with the exception that the aggressor was absent, was a significant stressor, likely involving social isolation stress. Although similar in Ctrl and AggE mice, immune cell infiltrates of liver, gallbladder, kidney, and stomach differed from each other in trends of rate of development and the impact of the stressor duration (S2 Fig.). Kidney lymphoplasmacytic interstitial infiltrates apparently worsened with time, while liver lymphoplasmacytic infiltrates lessened during stress-withdrawal. For organs with infiltrates that increased during stress-withdrawal, the trends suggest the possibility that the 10-day regimen might have been more damaging than the 5-day regimen, and/or the alterations could have been cumulative or developed more fully by 4 wks than by 1.5 wks.

### Elevated plasma proteins at 24 hrs post-stressor indicate damaged tissue in AggE mice

In plasma, we detected 41 proteins in the Rodent Multi-Analyte Profile v.2.0 Antigens (Myriad-RBM) panel at 24 hrs after the 5- and 10-day stressors. This analysis showed that haptoglobin and myeloperoxidase and serum amyloid P-component (False Discovery Rate *p*≤0.05 [16]) were significantly elevated in plasma of AggE over Ctrl mice at 24 hrs for the 10-day-exposed mice as well as for the combined group of 5-day- and 10-day-exposed mice (S2 File). These proteins are involved in responses to oxidative stress, damaged tissue, and inflammation. Myeloperoxidase is stored in azurophilic granules of neutrophils, oxidizes chloride ions to the potent bactericidal oxidant hypochlorous acid [17], and has been widely used as marker of inflammation in acute and chronic conditions, including cardiovascular disease [18]. Haptoglobin is an acute phase protein, increased during inflammation [19], which has been observed to rise in response to a number of psychological stresses as well as depression [20,21]. Serum amyloid P-component (SAP) is a member of the highly conserved pentraxin superfamily along with the classical acute-phase reactant, C-reactive protein (CRP); both SAP and CRP transcription are activated in response to endoplasmic reticulum stress [22]. SAP has anti-fibrotic effects [23] and binds molecular arrays such as DNA, chromatin, histones, and phosphoethanolamine-containing membranes, suggesting it functions in the clearance of late apoptotic cells [24].

Additionally, tissue inhibitor of metalloproteinases 1 (TIMP-1) was elevated in 10-day AggE over Ctrl mice (FDR corrected, *p* = 0.038), and matrix metalloproteinase-9 (MMP-9) was elevated in the 5-day-exposed over the 10-day-exposed mice (combined Ctrl and AggE groups). MMP-9 and matrix metalloproteinase family members are involved in the degradation of extracellular matrix in processes such as wound healing, and TIMP-1 is natural inhibitor of MMP-9, and the two proteins are markers consistently indicted in cardiovascular disease development and prognosis [25,26]. MMP-9 levels have been associated with direct effects of cortisol and norepinephrine (references in [27]), and independently predicted new cardiac events in patients with stable coronary disease [28], as well as having been independently associated with psychosocial factors in a normal middle-aged population [27]. Additionally, in our model, in heart tissues, MMP-2 and TIMP-1 transcripts were elevated in AggE mice at 24 hrs after the 5-day and 10-day stress regimens.

In contrast, granulocyte chemotactic protein-2 (GCP-2) and macrophage-derived chemokine (MDC) were elevated in Ctrl relative to AggE mice at 24 hrs, which may indicate a different trajectory of inflammation resulting from the stress of the Ctrl condition.

### Gut microbiota-derived metabolites in plasma of AggE mice were altered at both acute (24 hrs) and chronic (1.5&4 wks) time points

To explore metabolic alterations occurring at 24 hrs that potentially persisted out to 1.5 and 4 wks, we used random forests (RF) to classify AggE vs. Ctrl at 24 hrs and 1.5&4 wks (combined 5-day and 10-day groups). The resulting lists of the top 10% of metabolites with the greatest ‘mean decrease accuracy’ (MDA) at the acute and chronic time points were analyzed for overlaps [29]. Nine metabolites overlapped between the two lists (Table 2, *p* = 0.002, Fisher’s exact test). Five of the 9 overlapping metabolites were gut-derived metabolites: phenylpropionylglycine, phenol sulfate, hippurate, 3-phenylpropionate, and p-cresol sulfate. Three of these—3-phenylpropionate, phenylpropionylglycine, and hippurate—were elevated in AggE mice at both acute and chronic time points post-10-day regimen (t-test, *p* ≤ 0.05), and the acute and chronic time points of the combined 5- and 10-day group (t-test, *p* ≤ 0.05). These three metabolites are among the most elevated metabolites in AggE mice, being on average 6.7-fold elevated at 24 hrs and 2.8-fold elevated at 1.5&4wks.

**Table 2:** Metabolites that classify AggE vs. Ctrl mice at both acute and chronic time points.

	24 hrs			1.5&4 wks		
	MDA	FC	p-value	MDA	FC	p-value
Phenylpropionylglycine*	14.3	10	0.005	3.5	3.95	0.024
phenol sulfate*	14.2	1.27	0.090	5.9	0.77	0.143
Hippurate*	12.6	8.07	0.008	3	2.52	0.025
3-phenylpropionate (hydrocinnamate)*	7.9	2.16	0.021	7.3	1.97	0.015
p-cresol sulfate*	7.1	0.69	0.244	5.2	0.77	0.054
2'-deoxycytidine	22	1.64	0.001	2.1	1.13	0.152
palmitoyl sphingomyelin (d18:1/16:0)	13.5	1.26	0.007	15	1.11	0.015
creatinine	7.8	0.76	0.022	8.2	0.88	0.159
2-linoleoylglycerophosphocholine	6.8	0.83	0.183	6.1	0.91	0.277

MDA—‘mean decrease accuracy’ from random forests analysis

FC—fold change

*metabolites derived from gut microbiota

The metabolite 3-phenylpropionate is a known metabolic product of anaerobic bacteria [30]. Both hippurate and phenylpropionylglycine are derived from 3-phenylpropionate. Phenylpropionylglycine is the glycine conjugate of 3-phenylpropionate. Hippurate is a glycine conjugate of benzoate, derived from 3-phenylpropionate via catalysis by Medium-chain acyl-CoA dehydrogenase (MCAD) [31,32]. Hippurate has been found to be 17-fold lower in plasma of germ-free mice than conventional mice, and phenylpropionylglycine was detected exclusively in conventional samples [30]. Phenol sulfate and p-cresol sulfate have been shown to be reduced several hundred to 1000-fold in germ-free mice [30].

The remaining four metabolites identified by RF that overlapped between 24 hrs and 1.5&4 wks were apparently diverse. Notably, 2’-deoxycytidine was the best single classifier of AggE vs. Ctrl mice in the combined 5- and 10-day groups at 24 hrs using either distance-dependent K-nearest neighbor analysis (sensitivity = 90.9%, specificity = 81.8%) or rule-based machine learning.

### Gut microbiota-derived and other metabolites in plasma altered at either acute or chronic time points

In addition to the gut-derived metabolites identified by RF, pairwise and correlation analyses revealed additional gut-derived metabolites that were elevated in AggE at 24 hrs. Two of six metabolites that were “perfect classifiers” of AggE vs. Ctrl mice (i.e., levels were all higher or lower in AggE mice) at 24 hrs post-5-day stressor are metabolites of the gut microbiota; 2-(4-hydroxyphenyl)propionate and indolelactate. Both were elevated in AggE vs. Ctrl mice (combined 5- and 10-day groups). 2-4-hydroxyphenyl)propionate is a fecal bacteria fermentation product of tyrosine [33,34], which increased in feces of antibiotic-treated mice [35], and it also positively correlated in our dataset with indolelactate (r = 0.86), which is the other elevated perfect classifier. Indolelactate is a tryptophan metabolite produced by *Bifidobacterium* species [36], and was found to be at least 10-fold more abundant in urine of germ-free mice colonized with *Bifidobacterium longum* than in germ-free mice or mice colonized with *Bacteroides thetaiotaomicron* [37]. It was also 7-fold reduced in serum in a model of inflammatory bowel disease [38]. Additionally, 2-(4-hydroxyphenyl)propionate correlated with indolepropionate (r = 0.71). Indolepropionate has been identified in the plasma of conventional mice but not in germ-free mice [30].

Of the top 10%, or 33, highest MDA metabolites, 24 did not overlap between the 24 hr and 1.5&4 wk time points. Unique to the 24 hr time points were the additional gut-derived metabolites indolelactate and 2-(4-hydroxyphenyl)-propionate (also identified as perfect classifiers), and one of their correlating gut-derived metabolites, indolepropionate. Another gut-derived metabolite altered at 24 hrs was phenyllactate [39]. Thus, these gut-derived metabolites appear altered at the acute time, in addition to the gut-derived metabolites altered at both the acute and chronic times. Additionally, two fibrinogen cleavage peptides were highly ranked unique RF classifiers of AggE mice at the 24 hr time point. Fibrinogen cleavage products have been associated with inflammation [40].

The RF-classifying metabolites unique to 1.5&4wks included three plant-derived biochemicals, campesterol [41], piperidine [42] and daidzein [43]. Additionally, seven lysophospholipids were among the RF top-scoring metabolites at 1.5&4 wks and they were all elevated. These results suggest that AggE stress may have damaged the GI tract [44]. In addition to a likely altered gut microbiota, other changes could possibly include altered intestinal absorption and altered liver metabolism. The GI tract responds to stress and mediators of stress (including glucocorticoids and catecholamines) with disturbed motor activity, ulcerations, enteritis, and altered transepithelial ion transport [45–47], all of which can affect the composition of the microbiota [48]. Also, gut bacteria can respond directly to catecholamines with changes in growth and virulence gene expression [45]. Alterations in intestinal bacterial-host interactions have been suggested to cause liver injury [49].

### Metabolites of the major metabolic fuels in plasma were altered at acute and chronic time points

**Figure 2:**
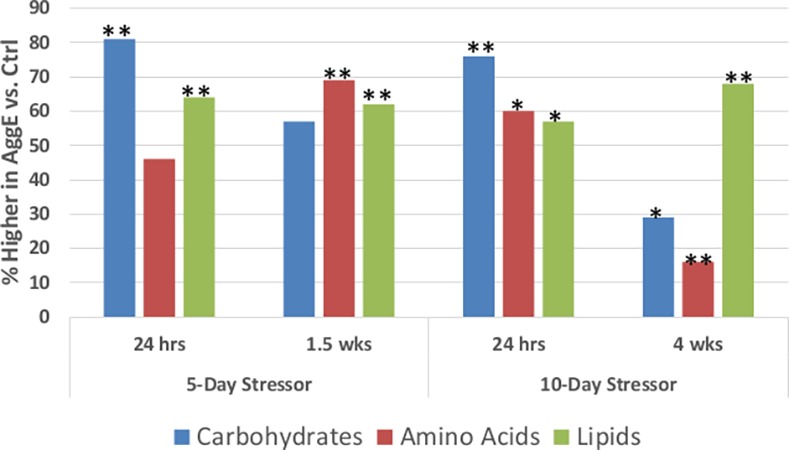
Percent of metabolites of metabolic fuels higher in AggE vs. Ctrl mice. The Carbohydrate, Amino acid and Lipid superpathways are comprised of 21, 98, and 134 metabolites, respectively. Asterisks indicate *p*≤0.05 (*) and *p*≤0.01 (**) for obtaining the percent elevated metabolites shown (exact binomial test).

The major metabolic fuels, carbohydrates, amino acids, and lipids differed in plasma levels dependent upon the stressor duration and the length of the stress-withdrawal period (Fig. 2). At 24 hrs after the 5- and 10-day stressors, 81% and 76% of the 21 carbohydrate metabolites were higher in plasma of AggE mice (binomial test, *p* = 0.003, post-5-day stressor, and *p* = 0.013, post-10-day stressor). But after withdrawal from the stress for 1.5 and 4 wks, only 57% and 29% of plasma carbohydrates were elevated, fewer than at 24 hrs (test of equal proportions: *p* = 0.182 and *p* = 0.005, respectively; and *p* = 0.002 for the combined 5-day and 10-day group).

Acutely elevated carbohydrate metabolites in plasma of AggE mice could possibly reflect the action of the primary stress mediators, epinephrine and corticosterone, on fuel storage and availability. Corticosterone mobilizes amino acids mainly from muscle, making more available in the plasma to enter gluconeogenesis in the liver to promote glucose and glycogen synthesis [50]. Thus, more glycogen is made available for glycogenolysis stimulated by epinephrine, which can release within minutes large amounts of glucose into the blood [50]. The rise in blood glucose in turn stimulates insulin secretion, but high levels of glucocorticoid make skeletal muscle and adipose tissue resistant to insulin’s stimulatory effects on glucose uptake and utilization [50]. Consistent with this scenario, at the acute time point, glucose level showed a trend to be elevated 1.2-fold in the 5-day 24 hr group (*p* ≤ 0.1) and 1.18-fold in the combined 5-day and 10-day 24 hr group (*p* ≤ 0.1). Also possibly consistent with this trend, 2-hydroxybutyrate, an indicator of insulin resistance and impaired glucose regulation [51–53], was 1.74-fold elevated in the combined group of 5- and 10-day AggE mice at 24 hrs (*p* = 0.007). However, changes in 2-hydroxybutyrate have also been related to the oxidative stress response in the liver and increased glutathione synthesis.

Conversely, the number of amino acid metabolites elevated in AggE mice at 24 hrs post-5-day stressor was not greater, but more of them were elevated at 24 hrs after the 10-day stressor (60% of 98 metabolites, *p* = 0.017). Of the major metabolic fuels, only amino acid metabolites appeared to differ between the 5-day-24 hr and 10-day-24 hr groups (proportion test, *p* = 0.063), coincident with habituation. More amino acid metabolites were also elevated in AggE mice at 1.5 wks, and significantly more than at 24 hrs (*p* = 0.001). The fewer plasma amino acid metabolites of AggE mice at 24 hours after the 5-day regimen may have possibly resulted from higher glucocorticoid signaling, resulting in depleted muscle capacity for mobilizing amino acids.

At 4 wks post-stressor, the great majority of amino acid metabolites, as well as carbohydrate metabolites, were lower in the plasma of AggE vs. Ctrl mice. Only 29% of carbohydrate and 16% of amino acid metabolites were higher in AggE than in Ctrl mice at this time point, while 68% of lipid metabolites were elevated in AggE.

A possible explanation for these trends is that at the 4 wk time point, when the allostatic load of AggE mice was evidently lowest, there may have been a return toward baseline of the sensitivity of hypothalamic-pituitary axis (HPA), as well as its effectors muscle and liver. In this scenario, the elevated lipid metabolites may have resulted in part from the situational cue of the aggressor during the partition test, which occurred less than one hour prior to the terminal bleed. This likely elicited a fear-related memory that activated the sympathetic-adrenomedullary axis, thus producing epinephrine, which activates hormone-sensitive lipase to mobilize lipids from adipose tissue. In contrast, glucocorticoid genomic programing of muscle and liver at 4 wks had likely returned nearer to baseline, and the fear-related memory only one hour before the terminal bleed may have been too short for slow glucocorticoid genomic changes to have taken full effect. The results of this could have been lower amino acid mobilization and gluconeogenesis, which would explain the low numbers of amino acid and carbohydrate metabolites in the plasma of AggE mice at 4 wks relative to the other time points when allostatic load was evidently higher.

Consistent with this scenario, plasma corticosterone trended higher at 4 wks relative to the earlier time points. In our unbiased metabolomic analysis (semi-quantitative LC-MS/MS analysis), corticosterone trended lower in AggE than in Ctrl mice at the two 24 hr points and at 1.5 wks, but trended higher in the AggE mice at 4 wks. Following up on this result, we performed a corticosterone ELISA on plasma samples from a replicate set of mice groups and got the same result; corticosterone trended lower in AggE mice at the two 24-hour points and at 1.5 weeks, but trended higher in AggE mice at 4 wks. Thus, two different assays performed on different plasma samples resulted in the same trend of relative corticosterone levels between AggE and Ctrl at four time points (*p* = 0.06 for this binomial distribution).

One possible explanation for these trends is that at both 24 hr time points and the 1.5 wks time point, the HPA had already become desensitized, potentially involving attenuated release of pituitary ACTH and/or reduced adrenal responsiveness [54]. However, at these earlier times of higher allostatic load, glucocorticoid-programing of muscle and liver may have been already put them into a heightened state for mobilizing amino acids and producing glycogen. But these heightened states could have diminished by 4 wks, although HPA responsiveness may have been restored. This could possibly explain the elevated corticosterone one hour after exposure to the situational cue of the AggE, despite carbohydrate and amino acid metabolites being lower in AggE mice at this time.

Another model based on the social defeat paradigm (in NMRI mice) found that repeated stress increased core body temperature (as in our model) and after one social defeat, corticosterone levels peaked at 30 min [55]. By one day after the single social defeat, corticosterone level was also increased, but not after the 4^th^ or 8^th^ defeat over 2 wks, while it was dramatically increased 24 hrs after the 12^th^ defeat after 3 weeks of experimentation [55]. Also in this study, mice after the 12^th^ defeat that were withdrawn from the stress for 9 days were sensitized to a novel stressor. The trend we observed is similar to this and suggests the possibility that the HPA axis was de-sensitized at 24 hrs and 1.5 wks and may have become re-sensitized by 4 wks.

Lipid metabolites in plasma of AggE mouse were elevated at all the time points. This effect could have been due to the rapid activation of hormone-sensitive lipase by adrenalin in adipose tissue to increase fat utilization for energy during the stress of the fear memory and afterwards. Elevated lipid metabolites could also result from chronic peripheral effects, including damage to and reprogramming of the organs that absorb, mobilize, and utilize the major fuels [56].

### Specific metabolite subpathways altered by AggE stress

**Figure 3:**
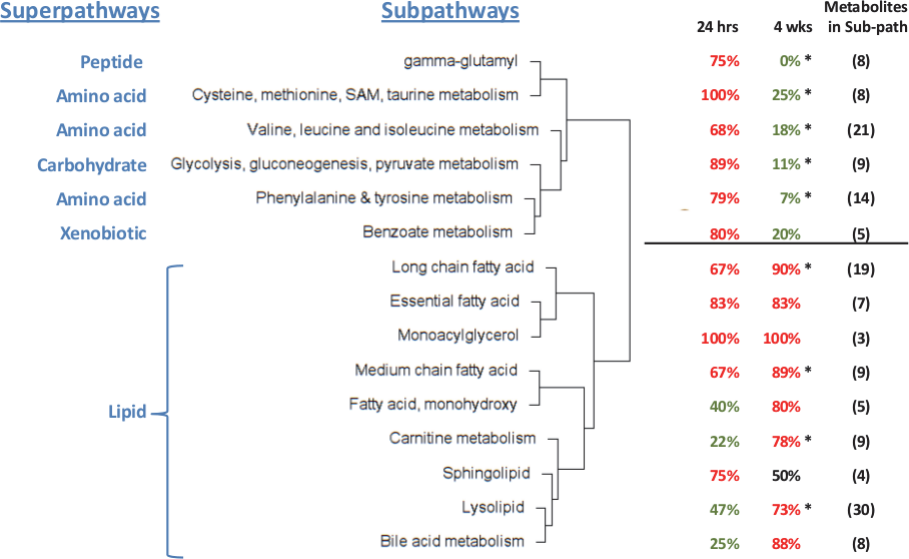
Dendrogram of 15 subpathways whose metabolites’ plasma levels changed coordinately. Shown is the hierarchical clustering of subpathways by mean Manhattan distance score, which describes the similarity in the direction of change (higher or lower) of metabolites of a subpathway in the eight comparisons of AggE vs. Ctrl and 24 hrs vs. 1.5 or 4 wks. Also shown for each subpathway is the percentage of metabolites higher (red) or lower (green) in AggE vs. Ctrl mice at 24 hrs and 4 wks after the 10-day stressor. The distance means for the lipid and non-lipid branches of the dendrogram were 3.1 (*p* = 1E-04) and 3.07 (*p* = 1E-04). Asterisks indicate the subpathways also determined by exact binomial test to have more metabolites lower or higher in AggE vs. Ctrl.

To assess possible coordinate regulation of metabolites related through chemical pathway or structure, we used a Manhattan distance method (see Methods section). This method identified 15 subpathways (of the 65 subpathways in our data set) whose metabolites were coordinately altered across the 8 comparisons of AggE vs. Ctrl and 24 hrs vs. 1.5 or 4 wks. These 15 subpathways were hierarchically clustered based on their metabolites’ distance means. This produced two large clusters. One consists of 9 subpathways of the lipid superpathway. The other consists of 3 subpathways under the Amino Acid superpathway and one subpathway under each of the Peptide, Carbohydrate and Xenobiotic superpathways (Fig. 3). The most distinct differences between these two clusters are the suppressed metabolites of the non-lipid subpathways and the elevated metabolites of the lipid subpathways at 4 wks.

**Figure 4:**
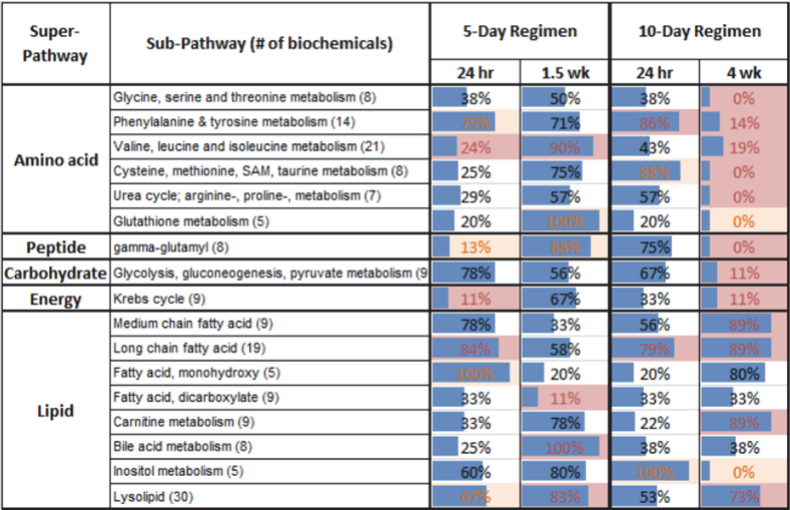
Percent of metabolites in subpathways elevated in AggE vs. Ctrl. Blue bars represent the percentage of all metabolites in the subpathway that were elevated in plasma of AggE vs. Ctrl mice. Red shading indicates *p* ≤ 0.05 and light orange shading indicates *p*≤ 0.1 (exact binomial test). Subpathways were included in the figure only if there was a significant difference at these *p*-values in one of the four AggE vs. Ctrl comparisons.

**Figure 5:**
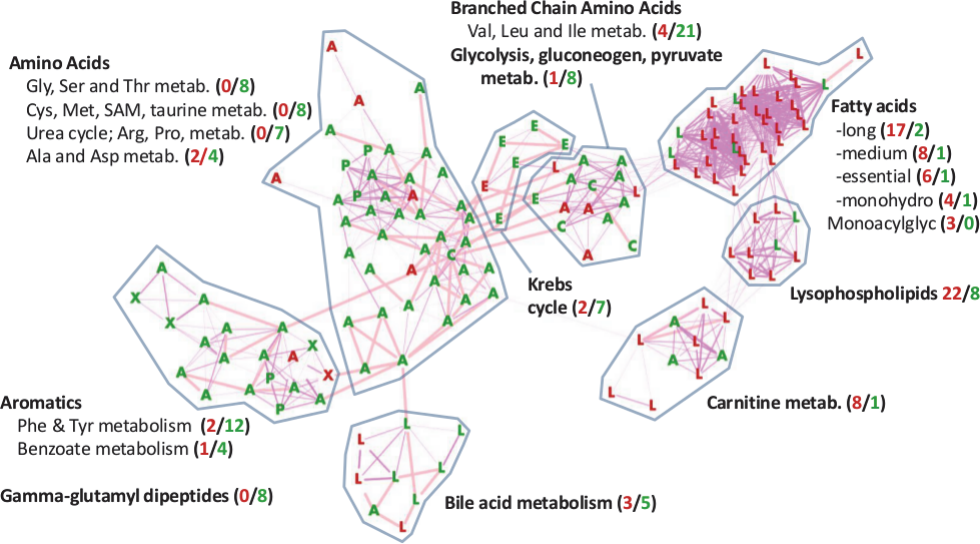
Higher levels of lipid and lower levels of amino acid metabolites in plasma of AggE vs. Ctrl mice at 4 wks after AggE-withdrawal. Shown are individual metabolites that were lower (green) or higher (red) in AggE vs. Ctrl mice at 4 wks post-AggE. Metabolites are shown for subpathways that had metabolites that were coordinately regulated (shown in Fig. 3) or that had significantly different numbers of higher or lower metabolites in AggE mice (shown in Fig. 4). The node labels indicate the superpathway designation of the metabolites: **A**, Amino acid; **C**, Carbohydrate; **L**, lipid; **E**, energy; **P**, Peptide; and **X**, xenobiotic. The metabolites are distributed using a force-directed, edge-unweighted algorithm in Cytoscape [89], with biochemical relationships shown as pink edges (KEGG) and structural similarities shown as violet edges (Tanimoto similarity >0.7). Metabolites for which a KEGG or PubChem Compound (CID) Identifier was available are shown. Numbers in parentheses indicate the number of elevated (red) and suppressed (green) metabolites in the subpathway, regardless of the availability of KEGG or CID identifiers. Subpathway labels are shown next to the groupings (by eye) where they were most abundant.

We also evaluated all of the 65 subpathways, including the 45 subpathways without significantly co-regulated metabolites, to identify subpathways with more AggE vs. Ctrl metabolites elevated or suppressed at any time point (exact binomial test). The most marked change was at 4 wks (Fig. 4) when many of the altered pathways were the same ones found with the Manhattan distance method. Additionally, the exact binomial test identified the subpathways ‘Glycine, serine and threonine metabolism’, ‘Urea cycle; arginine-, proline-, metabolism’, and ‘Krebs cycle’, as being suppressed in AggE vs. Ctrl mice at 4 wks after the 10-day stressor. The subpathways identified by both methods are shown overlaid onto the network of their structural and pathway relationships, which also depicts the relative levels of their metabolites in plasma of AggE vs. Ctrl mice (Fig. 5).

Overall, at 4 wks after the stressor, the elevated lipids of AggE mice plasma included several structural classes of fatty acids (medium and long chain, essential, and monohydroxy), lysophospholipids, carnitine metabolites and bile acids. Conversely, suppressed subpathways included ‘Glycolysis, gluconeogenesis, pyruvate metabolism’, ‘Krebs cycle’ and several subpathways of amino acid metabolism. This analysis is consistent with the superpathway analysis (major fuel metabolites) discussed above, in which carbohydrates and amino acids were suppressed and lipids were elevated in AggE vs. Ctrl mice at the 4 wk time point.

The relationships of these altered subpathways to each other, and to the stressor durations and stress-withdrawal periods are complex, but hypotheses can be inferred. For example, the five conjugated bile acids in plasma were all lower (*p* = 0.031, binomial test) in 5-day AggE mice at 24 hrs (by 1.32-fold on average), which could reflect higher sympathetic nervous system activity thus causing changes in the gut and liver at this time of high allostatic load. In contrast, after a 1.5 wk withdrawal from stress, all five conjugated bile acids were higher in AggE mice vs. Ctrl mice (5.9-fold average) as were the three unconjugated amino acids (i.e., 8 of 8 were elevated *p* = 0.004). Also, in the combined group of 5-day and 10-day mice, total bile acids in AggE plasma was 1.15-fold lower (6 of 8 lower) at 24 hrs and 3.12-fold higher (6 of 8 higher) than in Ctrl plasma at 1.5&4 wks. Elevated total serum bile acids is an indicator of liver disease and can reflect reduced hepatic clearance of bile acids from the hepatic portal blood. Additionally, bile acid metabolism can be regulated by the gut microbiota, and the bile acid tauro-muricholic acid is an antagonist of the farnesoid X receptor (FXR) [57].

### In liver, inflammation at 24 hrs and metabolic alterations at 4 wks are indicated by transcriptome analysis

Although lymphoplasmacytic infiltrates of livers of Ctrl and AggE mice showed similar levels (S2 Fig.), sub-histological alterations could have been present. We therefore analyzed differentially expressed genes (DEGs) of liver for enrichment in the ‘inflammatory response’ function annotation. More AggE vs. Ctrl DEGs (up- and down-regulated genes) were enriched in this function and with more significant *p*-values at 24 hrs than at 1.5 and 4 wks. Because the infiltrates only contribute transcripts to the tissue, we also analyzed up-regulated transcripts alone. These up-regulated transcripts in AggE mouse liver were enriched in ‘immune cell trafficking’ at 24 hrs, but not at 1.5 or 4 wks.

Upstream regulator analysis also indicated inflammatory changes of liver at 24 hrs, as the cytokines IL1B and TNF were predicted to be activated with z-scores greater than 2.0 (z-scores were 3.2 and 2.2; and *p*-values 2.04E-07 and 1.05E-06, respectively)[58]. Conversely at 4 wks these predicted cytokines trended toward being inhibited (IL1B: z-score -1.170, p-value 8.72E-04; TNF: z-score -0.303, p-value 1.77E-05). Of all the cytokines with a significantly predicted state of upstream activation for both 24 hr and 4 wk time points, 16 were predicted to be in an activated state (CCL2, CSF2, EDN1, IFNB1, IL1A, IL1B, IL17C, IL18, IL22, IL23A, IL27, IL33, LIF, OSM, TNF, and TNFSF12) and 2 were predicted to be in an inhibited state (IL3, TNFSF12). All 16 with an activated state were predicted for the 24 hr samples, and the two activators with an inhibited state were predicted for the 4 wk samples.

Of all upstream activators at any time point, the topmost one predicted for the AggE vs. Ctrl DEGs was ‘lipopolysaccharide’ (smallest *p*-value) and it was predicted to be highly activated at the 24 hr points (z-scores were 4.4 and 3.1; *p*-values 5.42E-06 and 4.5E-03, respectively, for post-5- and 10-day stressors), but trended toward being inhibited at 1.5 and 4 wks (z-scores -1.9 and -1.7; p-values 2.83E-01 and 9.70E-06, respectively, for 5- and 10-day stressors).

At 24 hrs post-stressor, despite no detectable difference in liver lymphoplasmacytic infiltrates between AggE and Ctrl mice, the gene set enrichment analysis indicated that the AggE livers were more inflamed than the Ctrl livers. Additionally, well known stress response genes were among the highest up-regulated genes in AggE mouse liver at this acute time point (e.g., lipocalin 2, metallothioneins, serum amyloid proteins). These data suggest sub-histopathological liver damage was an early effect of the AggE stress.

In contrast to the indicated inflammation at 24 hrs post-stressor, a different picture emerged at 4 wks post-stressor. Downstream function analysis in the ‘nutritional disease’ category showed significant enrichment in ‘adult-onset obesity’. The 42 enriching genes in ‘nutritional disease’ included 26 that were enriched in ‘weight gain’. This is consistent with the body weight gain of AggE vs. Ctrl mice that steadily increased up to the 10^th^ day of the stressor regimen (data not shown). In the category ‘hepatic system development and function’, significant enrichment was found in ‘abnormal morphology of liver’ (liver, parenchyma, and hepatocytes; B-H p-value = 4.49E-02, 24 genes). In the ‘hepatic system development and function’ category overall, 38 genes were enriched. The top six canonical pathways associated with these genes included ‘PPARα/RXRα activation’, ‘aryl hydrocarbon receptor signaling’ and ‘PPAR signaling’. All were predicted to be activated in AggE-mice. Activation of the PPAR pathways is consistent with the elevated levels of their ligands (free fatty acids) in plasma [59] at 4 wks.

Activation of ‘aryl hydrocarbon receptor signaling’ has been associated with refractoriness to a second LPS challenge, thought to be part of a “disease tolerance” mechanism [60,61]. Thus, one possibility is that chronically activated ‘aryl hydrocarbon receptor signaling’ could have been the consequence of liver exposed to gut bacteria. Notably, LPS (lipopolysaccharide) was the topmost predicted activated upstream activator of the AggE-altered DEGs at 24 hr post-stressor.

Further analysis of the 4 wk downstream enrichments of AggE vs. Ctrl DEGs included only functional annotations with both a significant *p*-value for the overlap as well as a significant activated state. This resulted in the function annotations: ‘conversion of lipid’, ‘biosynthesis of nucleoside triphosphate’, ‘ascites’, ‘differentiation of granulocytes’, ‘differentiation of myeloid cells’, and ‘contact growth inhibition of leukocytes’. The immune cell functions involving differentiation and growth inhibition appear to be consistent with the lymphoplasmacytic infiltrates that persisted at the chronic time points but trended toward a lower grade at 1.5 and 4 wks than at 24 hrs (S2 Fig.).

To look further for pathways altered chronically by AggE, we performed upstream regulator analysis on the 4-wk DEGs, focusing on upstream regulators with both significant *p*-values and z-scores. Four were predicted to have an inhibited state in AggE mouse liver: RARG (CD437), Map4k4, HOXC8, and D-glucose. Conversely, the activators with a predicted activated state totaled to 12: SREBF1, SREBF2, ESRRA, ESRRG, CHUK, phenobarbital, TP53, SCAP, PPARG, mono-(2-ethylhexyl)phthalate, sulforafan, and TO-901317. Several of these are plausibly related through regulatory pathways (e.g., mono-(2-ethylhexyl)phthalate, PPARG, PPARA, SCAP, SREBF1, and SREBF2; all found using Ingenuity Pathway Analysis (IPA)). The 12 activated upstream regulators (excluding p53) plus their target molecules summed to 109 molecules (S3 File). Analysis of their overlap with canonical pathways showed that the top 10 canonical pathways (Table 3) generally predicted *activation* of lipoprotein metabolism, fatty acid uptake and metabolism, lipogenesis, and phase I and II metabolism of lipids and xenobiotics; and *inhibition* of cholesterol and bile acid synthesis and transport, as well as glucose uptake and gluconeogenesis.

**Table 3:** Topmost 10 canonical pathway enrichments at 4 wks post-stressor.

Canonical Pathway Name (genes enriched)*	Predicted downstream effects
***LPS/IL-1 Mediated Inhibition of RXR Function*** (ALDH9A1, CYP4A11, GSTP1, GSTT1, JUN, NR0B2, SLC27A3, SREBF1)	Inhibition of cholesterol and lipid metabolism and transport; activation of Phase I metabolizing enzymes->lipid and xenobiotic metabolism
***PPARa/RXRa Activation*** (ACAA1, CHUK, CYP2C18, GPD1, IRS1, JUN, NR0B2, TGFBR1)	Activation of fatty acid oxidation, fatty acid uptake, lipoprotein metabolism, mitochondrial and peroxisomal beta-oxidation.
***Oxidative Phosphorylation & Mitochondrial Dysfunction*** (ATP5B, ATP5D, COX6A2, NDUFA10, NDUFB5, SDHA, SDHB, UQCRC1)	Activation
***NRF2-mediated Oxidative Stress Response*** (ACTA2, EPHX1, FTL, GCLM, GSTP1, GSTT1, JUN)	Activation of phase I and II metabolizing enzymes and transport of xenobiotics and metabolites
***FXR/RXR Activation*** (CYP19A1, CYP8B1, FOXA2, NR0B2, PPARG, SDC1, SREBF1)	Inhibition of gluconeogenesis, increased lipogenesis and lipid metabolism
***Glucocorticoid Receptor Signaling*** (CCL11, CEBPA, CHUK, CSN2, JUN, TGFBR1)	Inconclusive
***Aryl Hydrocarbon Receptor Signaling*** (ALDH9A1, CDKN1B, GSTP1, GSTT1, JUN, NR0B2)	Activation of phase I and phase II metabolizing enzymes
***Superpathway of Cholesterol Biosynthesis*** (DHCR7, EBP, FDPS, HADHA, NSDHL)	Activation of cholesterol synthesis
***Type II Diabetes Mellitus Signaling*** (CHUK, IRS1, PPARG, SLC27A3, SLC2A2)	Reduced glucose entry into hepatocytes.

* Upstream activators (p≤0.05) that additionally had a predicted activated activation state (z-score >2) totaled to 12. These activators and their target molecules totaled to 109 molecules and were analyzed for overlaps with the shown canonical pathways.

Consistent with our data, chronic psychosocial stress has been show to induce lipid dysregulation, intrahepatic accumulation of triglycerides, indicators of metabolic syndrome [62,63] as well as hepatic oxidative stress and inflammation [64].

### Limitations in the Research Design

Our unbiased metabolomics data showed high *q*-values for pairwise comparisons of metabolite levels between Ctrl and AggE is likely due to the small sample size of 5 or 6 mice per group and the known highly variable stress-response of even inbred mice. To overcome this shortcoming, we applied a battery of statistical tests, including analyzing metabolite groupings constructed based on KEGG and HMDB, and the internal knowledge base of Metabolon, Inc.

### Conclusions and Hypothesis

The repeated stress for 5 or 10 days altered multiple organ systems acutely, and chronic systemic changes were also evident. Acutely at 24 hrs post-stressor, heart (frank lesions) and liver (transcriptomic changes) tissues showed evidence of inflammation, plasma protein levels indicated tissue damage, and the plasma levels of metabolites derived from the gut microbiota were altered. Chronically at 4 wks post-stressor, levels of gut-derived metabolites were altered, hyperlipidemia was evident, and liver transcriptomics indicated activation of xenobiotic and lipid metabolism. These data provide clues for ongoing efforts to understand and identify molecular indicators of physiologic responses to acute and chronic stressors that have potential to predict probable PTSD and monitor its persistence.

The altered plasma levels of gut-microbiota-derived metabolites in AggE mice may have resulted from the well-known effects of stress mediators on the GI tract to induce changed motor activities, ulcerations and enteritis [45,46] and changed gut microbiota composition [48,65]. Prolonged stress has been shown to produce low-grade intestinal inflammation [66], and inflammation can increase translocation of commensal bacteria across the epithelium that escape from local mesenteric lymph nodes, thus reaching the liver [49]. The liver’s ability to act as a firewall against systemic exposure to gut bacteria could be compromised by intense stress, and adrenergic receptor activation is known to constrict the hepatic artery and microvasculature, reducing total hepatic blood volume [67]. Social disruption stress has been reported to induce translocation of bacteria from the lumen of the intestines to lymphoid tissue of the body interior, and to increase the percent of livers with viable bacteria [68] as well as circulating cytokines [46,48]. Such stress-induced alterations could have contributed to the altered liver gene expression we observed.

Stress mediators simultaneously affect multiple organ systems. Simultaneous perturbations of closely cooperating systems like gut and liver could synergize to produce organ injury which leads to chronic changes. For example, liver disease resulting from bile duct ligation can cause low-grade intestinal inflammation and histological damage to intestine, increasing the probability of intestinal microbes translocating into the vasculature as well as reducing the ability of liver to clear them [49]. Also, patients with mostly asymptomatic liver steatosis or nonalcoholic steatohepatitis (NASH) had increased serum IgG and IgA responses against aerobic and anaerobic intestinal commensal bacteria. Additionally, these antibody responses discriminated the diagnoses from healthy controls [49]. Lastly, dysbiosis of intestinal microbes has been shown to precede or trigger liver damage [49,69,70].

The above findings collectively suggest the hypothesis that repeated stress effects on multiple organ systems can synergize to result in chronically altered gut-microbiota-derived metabolites, hyperlipidemia, and liver gene expression changes. Such interactions of stress effects among multiple organ systems might underlie reports of associations of PSTD with gastrointestinal complaints, metabolic syndrome, hypertension, hyperlipidemia, obesity, and cardiovascular disease [71–75].

## Methods

### Ethics statement

Research was conducted in compliance with the Animal Welfare Act, and other Federal statutes and regulations relating to animals and experiments involving animals and adheres to principles stated in the Guide for the Care and Use of Laboratory Animals (NRC 2011) in facilities that are fully accredited by the Association for the Assessment and Accreditation of Laboratory Animal Care, International. The protocol was approved by the Institutional Animal Care and Use Committee (IACUC) of Walter Reed Army Institute of Research, Silver Spring, MD, MedStar Health Research Institute, Washington DC and Animal Care and Use Review Office (ACURO), US Army Medical Research and Materiel Command (USAMRMC).

### Mice

All animal experiments were approved in writing by the Institutional Animal Care and Use Committee (IACUC) of the United States Army Medical Research and Materiel Command and the Medstar Research Institute (Protocol Number: 2011–010) and were conducted in compliance with the Animal Welfare Act, and other Federal statutes and regulations relating to animals and experiments involving animals, adhering to principles stated in the Guide for the Care and Use of Laboratory Animals (NRC 2011) in facilities fully accredited by the Association for the Assessment and Accreditation of Laboratory Animal Care, International. All mice were purchased from Jackson Laboratory, Bar Harbor, ME, USA. All mice had free access to food and water and were kept in a temperature-controlled room (21 + 2°C) on reverse 12/12 h light/dark cycle (lights off at 06:00 AM).

**Aggressor mice**. The SJL albino male mice (5 to 6 weeks old and weighing 30–35 g when purchased), which are characterized by extreme aggression in males [76], were housed individually in polycarbonate cages (48 X 27 X 20 cm) for one month prior to the experiment to induce aggressiveness due to isolation. They were then trained to attack intruders as described [11].

**Subject mice**. Male C57BL/6J mice (5 to 6 weeks old weighing 20–25 g) were singly housed, in a different room from the aggressor mice under the same environmental conditions, for one week prior to and during the stress regimens, and afterward during the 1.5 or 4 wk stress-withdrawal periods.

**Control mice**. Control mice were housed identically to subject mice.

### Aggressor exposure

Aggressor exposure (AggE) sessions followed a modified “cage-within-cage resident-intruder” protocol. For aggressor exposures, subject mice were placed in a wire mesh cage (17.5 X 14 X 7.5 cm) that was put inside the aggressor´s large plastic home cage (48 X 27 X 20 cm) for 6 hrs as described [11]. During the 6 hrs, mice were randomly placed in physical contact with the aggressor mouse 3 times, each for 1 min. or 10 strikes (whichever came first). The 6-hr sessions were repeated daily for either 5 or 10 consecutive days. In a separate room, control mice were sham-AggE using the same cage-within-cage environment and food deprivation on the same 6-hr daily regimen, but with fresh bedding and without any aggressor present. Aggressor mice were provided food and water *ad libitum* during the 6-hr “cage-within-cage” sessions. After each 6-hr session, AggE and sham-AggE (Ctrl) mice were single-housed in their home cages with *ad libitum* food and water until the next session. Twenty-four hours after the 5 and 10 days of daily 6-hr sessions, half of each population was randomly chosen and Ctrl and AggE were euthanized alternately. The other half of the population continued to be single-housed in their home cages until they were euthanized at 1.5 weeks (5-day AggE) or 4 weeks (10-day AggE).

One hour prior to the terminal bleed, mice were subjected to a partition test to evaluate behaviors in response to the situational reminder of the aggressor’s presence. Each subject mouse was placed into an aggressor’s home-cage on the other side of a partition that blocks physical interaction between the mice but permits passage of sensory cues. Subject mouse were then video recorded for five minutes for behavioral evaluations [11].

### Tissue collection and processing

Mice were euthanized by cervical dislocation. Ctrl and AggE mice were alternately euthanized and processed for tissue collection immediately after euthanasia. Plasma, liver and duodenum were frozen immediately until analysis. Heart, stomach, liver, gallbladder, and kidney were processed for histopathology.

Blood was collected from heart with a 20 gauge needle first rinsed with sodium citrate, and was transferred to tubes containing 50 μl of 0.015 *M* buffered sodium citrate. Tubes were mixed gently three times and then centrifuged. Plasma was transferred to a new tube and centrifuged again. The resultant clean and clear plasma was collected, immediately placed on dry ice, kept frozen, and shipped frozen to Metabolon, Inc. (Durham, NC), where the frozen plasma was immediately stored at -80°C.

For liver gene expression analysis, liver was weighed before thawing and Trizol^TM^ was added prior to homogenization and RNA isolation according to the manufacturer’s recommendations (Invitrogen, Grand Island, NY). The RNA was cleaned using the RNAeasy mini column (Qiagen). Extracted RNA from all samples was stored -80°C. RNA quality and quantity were assayed using a Nanodrop ND-1000 spectrometer (Thermo Fisher, Wilmington, DE). RNA from each sample was assessed for purity by A260/280 ratios. To determine the integrity of rRNA, 1 μL of eluate from each sample was analyzed using the Agilent 2100 Bioanalyzer or Agilent 2200 Tapestation (Agilent Technologies, Inc., Santa Clara, CA) according to the manufacturer’s instructions.

### Plasma metabolomics

Unbiased metabolomic profiles of plasma samples were obtained using ultrahigh performance liquid chromatography/tandem mass spectrometry (UHPLC/MS/MS) and gas chromatography/ mass spectrometry (GC/MS [77,78]. MS/MS data were searched against a library of purified standards or recurrent unknown entities [77], and the resulting named metabolites were used for our analysis.

Samples were extracted to remove protein and dislodge small molecules bound to protein or physically trapped in the precipitated protein matrix. Extracts were split for analysis on the gas chromatography (GC) and liquid chromatography (LC) platforms.

Sample extracts for LC/MS (LC/MS, LC/MS/MS) were split into two aliquots, dried, and then reconstituted in acidic or basic LC-compatible solvents, each of which contained 11 or more injection standards at fixed concentrations. One aliquot was analyzed using acidic positive ion optimized conditions and the other using basic negative ion optimized conditions in two independent injections using separate dedicated columns. Extracts reconstituted in acidic conditions were gradient eluted using water and methanol (both containing 0.1% formic acid) while the basic extracts, which also used water/methanol, contained 6.5 mM ammonium bicarbonate. The MS analysis alternated between MS and data-dependent MS^2^ scans using dynamic exclusion. The system was based on a Waters ACQUITY UPLC and a Thermo-Finnigan LTQ mass spectrometer, which consisted of an electrospray ionization (ESI) source and linear ion-trap (LIT) mass analyzer.

Samples destined for GC/MS analysis were re-dried under vacuum desiccation for a minimum of 24 hs prior to being derivatized under nitrogen using bistrimethyl-silyl-triflouroacetamide (BSTFA). The GC column was 5% phenyl and the temperature ramp was from 40° to 300°C during a 16 minute period. Samples were analyzed on a Thermo-Finnigan Trace DSQ fast-scanning single-quadrupole mass spectrometer using electron impact ionization. The instrument was tuned and calibrated for mass resolution and mass accuracy on a daily basis. The information output from the raw data files was automatically extracted.

For Accurate Mass Determination and MS/MS fragmentation (LC/MS), (LC/MS/MS), the LC/MS was based on a Waters ACQUITY UPLC and a Thermo-Finnigan LTQ-FT mass spectrometer, which had a linear ion-trap (LIT) front end and a Fourier transform ion cyclotron resonance (FT-ICR) mass spectrometer backend. For ions with counts greater than two million, an accurate mass measurement could be made on the parent ion as well as fragments. The typical mass error was less than 5 ppm. Ions with less than two million counts require a greater amount of effort to characterize. Fragmentation spectra (MS/MS) were typically generated in data dependent manner. Metabolon’s proprietary visualization and interpretation software was used to confirm the consistency of peak identification among samples.

Compounds were identified by comparison to purified standards or recurrent unknown entities. The purified standards consisted of more than 1800 commercially available compounds. Match to the specific compound or an isobaric entity was indicated by the combination of chromatographic properties and mass spectra. Additional entities were identified as recurrent entities (both chromatographic and mass spectral) and currently remain unidentified.

Superpathways and subpathways were constructed from information available in public databases (e.g., KEGG and HMDB and textbooks) and Metabolon’s internal knowledge base. They generally reflect structural and substrate-product relationships and/or the biology of the gut microbiota. These superpathways and subpathways were constructed prior to the statistical analyses.

**Corticosterone ELISA**. Blood collected in tubes containing 50 μl 0.105 *M* buffered sodium citrate was centrifuged to separate plasma that was stored at -80°C until the assay. The ELISA was based on a competitive enzyme immunoassay using a combination of specific antibodies to corticosterone and HRP-labeled corticosterone (Kamiya Biomedical Company, Seattle, WA).

### Plasma Proteins

Blood samples were collected in plastic tubes containing buffered sodium citrate (3.2%) and were centrifuged to separate plasma. Seventy μl of samples were shipped to Rules-Based Medicine, Inc. (Austin, TX). All samples were stored at -80°C until tested and were analyzed for Rodent Multi-Analyte Profile (MAP) v.2.0 Antigens (Myriad-RBM) using Luminex technology.

### Liver microarray gene expression analysis

mRNA was extracted from liver samples of 4 to 6 mice from each of the eight different time/treatment groups and were processed for dual dye microarrays (Whole Mouse Genome Microarray Kit, Agilent Technologies, Inc., CA) following the vendor’s protocol. Purified RNA was labeled with Cy-5 and the reference RNA (Agilent Technologies, Inc.) with Cy-3. The samples were simultaneously hybridized to Agilent 4 x 44k slides (platform number: 14868) for 17 h at 55°C. After hybridization, slides were processed in a series of washes and scanned using an Agilent DNA microarray scanner. Features were extracted using the default setting of the Feature Extraction software v.10.7 (Agilent Technologies, Inc.). The resulting expression data files were analyzed using custom R scripts. Arrays were background corrected, individually LOWESS-normalized [79], and quantile-normalized across all arrays. Probes were then filtered to include only those associated with a REFSEQ gene symbol (~26.5k out of 45k). The remaining probes were compared between groups using a moderated t-statistic with Benjamini*–*Hochberg false discovery rate (FDR) correction. The microarray data was submitted to the Gene Expression Omnibus (GEO). This can be searched using the Platform ID: GPL4134, Series: GSE58275.

### Quantitative Real-time PCR

Microarray results were validated using qPCR primer assays (SABiosciences, Frederick, USA) using Applied Biosystems 7900 systems. The RNA of each mouse was converted into cDNA using the RT^2^ First Strand Kit (SABiosciences, Frederick, USA). This cDNA was then added to the RT^2^ SYBR Green qPCR Master Mix (SABiosciences, Frederick, USA). All steps were done according to the manufacturer’s guidelines. The data were analyzed using ∆∆ct method [80] using 18S ribosomal RNA, ßactin and Ubiquitin C as housekeeping genes. Validation of microarray data for selected genes is shown in S3 Fig.

### Histopathology

Organ samples were collected from euthanized control and AggE mice and perfused in ice-cold 4% paraformaldehyde. Tissues were then stained, sectioned to 5 μm thickness, and mounted. A board-certified veterinary pathologist, blinded from the animal condition, identified and analyzed the samples using brightfield optics. Histologic findings were scored as normal, minimal, mild, moderate, and marked, as well as focal, multifocal and diffuse.

### Statistical analysis

Levels of metabolites or proteins in plasma between mouse groups were compared using pairwise methods including Welch’s t-test and ANOVA. Random forests analysis was used to identify metabolites that classify Ctrl and AggE mouse groups. The groups receiving the 5-day and 10-day stressors were analyzed individually and combined. We also used single-node decision trees (Gini index and Information Gain (Kullback–Leibler divergence)), Chi-square test, Kolmogorov-Smirnov test, and an in-house procedure that examines samples with near-extremum intensities, which is a simple classifier that uses a leave-one-out nearest-centroid procedure for classification. The five top scoring metabolites from the methods were selected as candidate discriminators and were correlated with each other. Those that correlated with a Pearson’s correlation coefficient > 0.7 were placed within one category, and only the metabolite within each category with the largest maximum intensity was evaluated further. These metabolites were correlated with all other metabolites to identify those potentially belonging to related metabolite pathways (Pearson’s correlation coefficient > 0.7, *p*-value of 0.024 for any comparison). All metabolites that were exact separators by Gini index and the remaining metabolites, plus those that correlated with them, were used in exhaustive searches with distance-dependent K-Nearest Neighbor (DD-KNN) and rule-based classifiers to identify sets of metabolites that best discriminate Ctrl from AggE mice.

Comparisons of up vs. down metabolites in Ctrl vs. AggE at each time point were done using a two tailed binomial exact test against the null hypothesis of a 50% up and 50% down, as implemented in the R binom.test function [81–83]. Comparisons of differences in the proportion of up vs. down metabolites between time points were done using a two-tailed difference of proportions test against the null hypothesis of equal proportions, as implemented in the R prop.test function [83–87].

A Manhattan distance method was used to identify subpathways having metabolites that were altered similarly among the eight simple comparisons of AggE vs. Ctrl and 24 hrs vs. 1.5 or 4 wks. Each metabolite of a subpathway was assigned either a 0 or a 1 for having its mean plasma level higher or lower in each comparison. Here, the actual direction of regulation is not important, only the pattern relative to other metabolites. To get the average distance of metabolites in a subpathway, each metabolite of a subpathway was compared pairwise with every other metabolite of the pathway. Two metabolites with the same 1’s and 0’s in all eight comparisons (same pattern of regulation across experimental conditions) have a distance of zero. Two metabolites with different 1’s and 0’s in all eight comparisons have a distance of 8. The total up vs. down Manhattan distance across all pairs of metabolites within a subpathway was tested against the null distribution of analogous distances created by taking 10,000 random samplings from all the metabolites of the number in the subpathway of interest. An approximate (slightly conservative) *p*-value was then calculated as (b+1)/(m+1), where b is the number of sample distances greater than the actual subpathway distance and m is the number of random samplings (10,000), as described in [88].

**Gene Enrichment Analysis**. Data were analyzed using causal analysis approaches in Ingenuity Pathway Analysis (IPA, Ingenuity Systems, Inc., Redwood, CA) to infer upstream biological causes and probable downstream biological effects, using enrichment score (Fisher’s Exact Test *p*-value) to measure overlap between observed and predicted gene sets, and z-score to match observed and predicted up/down regulation patterns [58].

## Supporting Information

S1 Fig Cardiac histopathology of AggE mice. Histopathology was scored as normal, minimal, mild, moderate, as well as focal, multifocal and diffuse. Number of mice evaluated: 5 Day-24 hrs: Ctrl = 7; AggE = 8. 5 Day-1.5 wks: Ctrl = 5; AggE = 5. 10 Day-24 hrs: Ctrl = 11; AggE = 11. 10 Day-4 wks: Ctrl = 5; AggE = 5.
(TIFF)

S2 Fig Histology of liver, gallbladder, stomach and kidney. Histology was scored as normal, minimal, mild, moderate, as well as focal, multifocal and diffuse. Number of mice evaluated: 5 Day-24 hrs: Ctrl = 7; AggE = 8. 5 Day-1.5 wks: Ctrl = 5; AggE = 5. 10 Day-24 hrs: Ctrl = 11; AggE = 11. 10 Day-4 wks: Ctrl = 5; AggE = 5.
(TIFF)

S3 Fig Validation of gene expression changes observed in microarray experiments. (TIFF)

S1 File Unbiased plasma metabolomics analysis. (XLSX)

S2 File Analysis of plasma proteins. (XLSX)

S3 File Upstream regulators plus their target molecules at 4 wks. (XLSX)

S1 Table Summary of altered metabolites. (TIFF)

S2 Table Liver gene enrichment suggests inflammation at 24 hrs post-5-day AggE was resolved at 24 hrs post-10-day AggE. (TIFF)
