# Supplementary figures and images for: Acute and Chronic Plasma Metabolomic and Liver Transcriptomic Stress Effects in a Mouse Model with Features of Post-Traumatic Stress Disorder
Source: PLoS One. 2015 Jan 28;10(1):e0117092. doi: 10.1371/journal.pone.0117092 (PMC4309402; doi:10.1371/journal.pone.0117092)

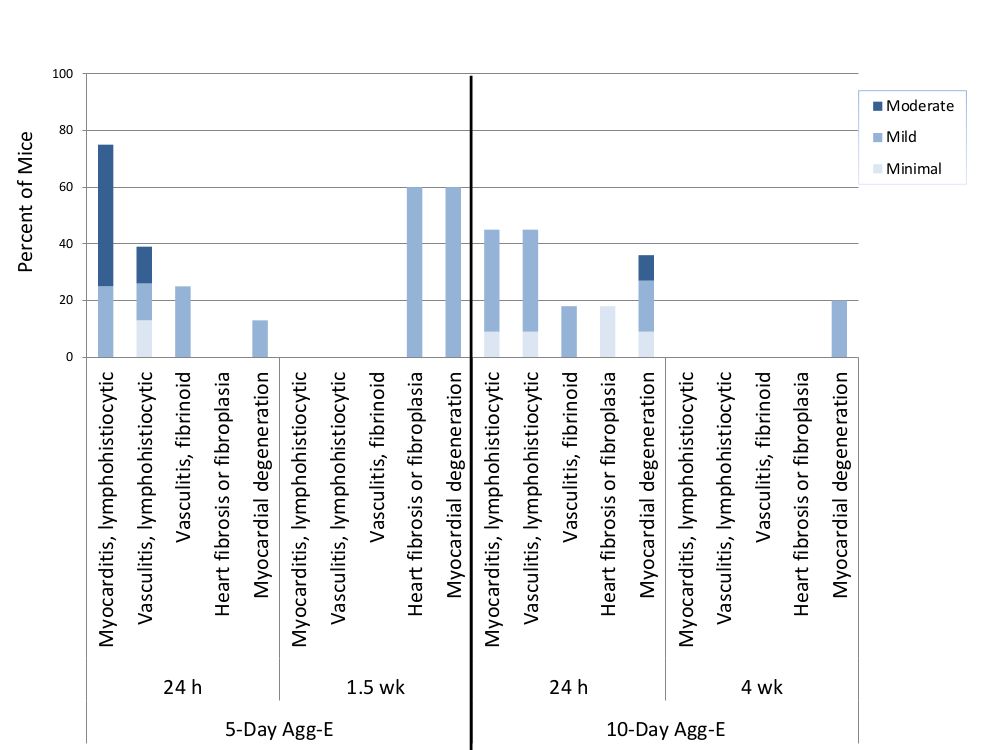

Supplement: S1 Fig — Histopathology was scored as normal, minimal, mild, moderate, as well as focal, multifocal and diffuse. Number of mice evaluated: 5 Day-24 hrs: Ctrl = 7; AggE = 8. 5 Day-1.5 wks: Ctrl = 5; AggE = 5. 10 Day-24 hrs: Ctrl = 11; AggE = 11. 10 Day-4 wks: Ctrl = 5; AggE = 5. (TIFF) [file pone.0117092.s001.tiff]

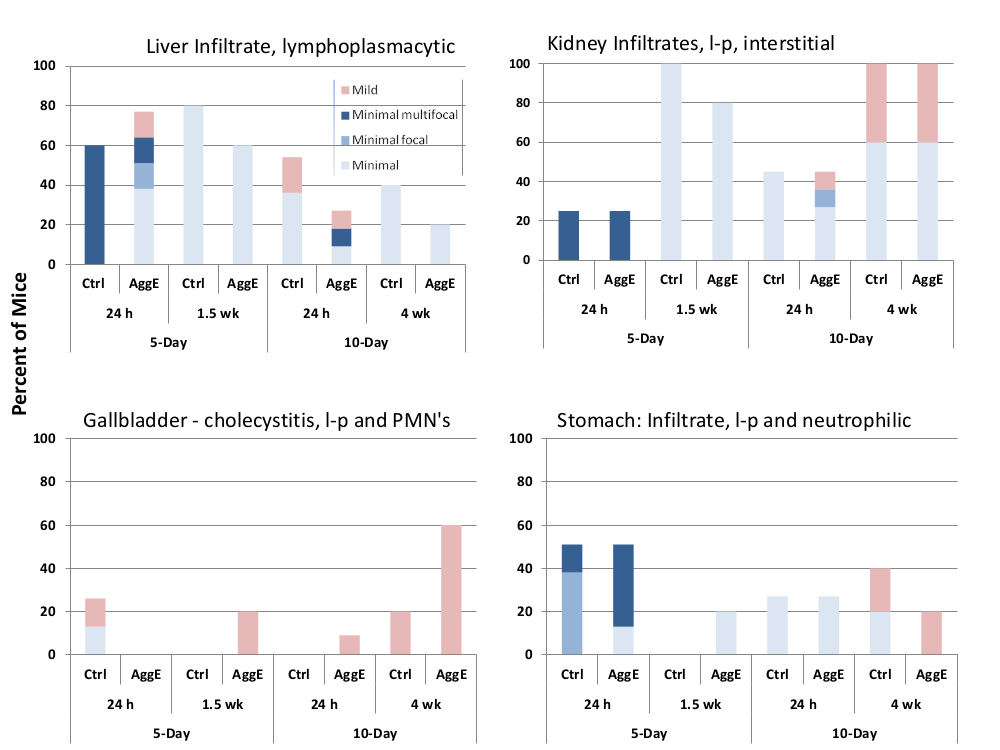

Supplement: S2 Fig — Histology was scored as normal, minimal, mild, moderate, as well as focal, multifocal and diffuse. Number of mice evaluated: 5 Day-24 hrs: Ctrl = 7; AggE = 8. 5 Day-1.5 wks: Ctrl = 5; AggE = 5. 10 Day-24 hrs: Ctrl = 11; AggE = 11. 10 Day-4 wks: Ctrl = 5; AggE = 5. (TIFF) [file pone.0117092.s002.tiff]

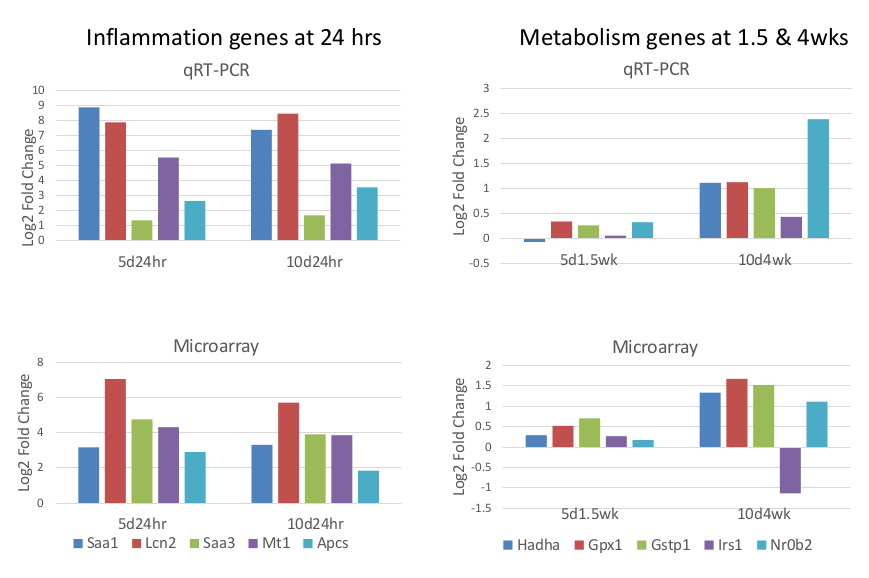

Supplement: S3 Fig — (TIFF) [file pone.0117092.s003.tiff]

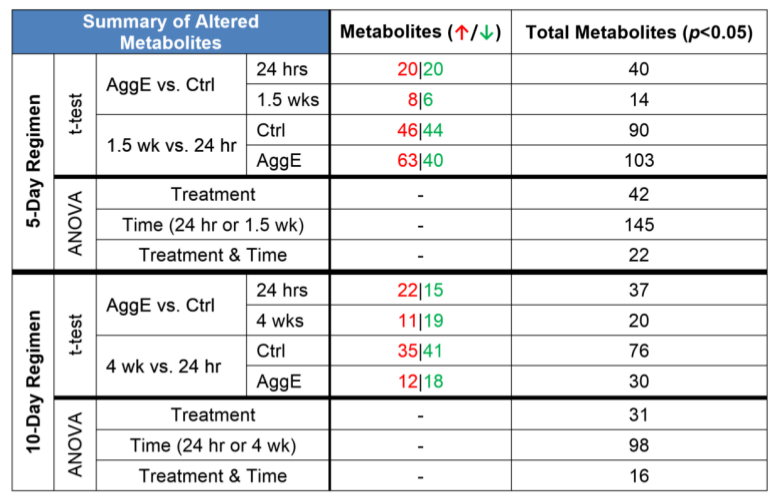

Supplement: S1 Table — (TIFF) [file pone.0117092.s007.tiff]

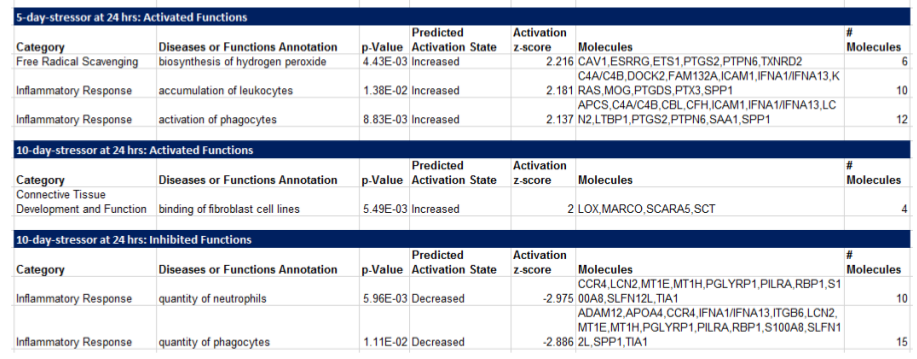

Supplement: S2 Table — (TIFF) [file pone.0117092.s008.tiff]
